# Single-Cell Analysis Reveals the Role of the Neuropeptide Receptor FPR2 in Monocytes in Kawasaki Disease: A Bioinformatic Study

**DOI:** 10.1155/2022/1666240

**Published:** 2022-06-01

**Authors:** Tengyang Wang, Guanghua Liu, Xiaofeng Guo, Wei Ji

**Affiliations:** ^1^Fujian Branch of Shanghai Children's Medical Center Affiliated to Shanghai Jiaotong University School of Medicine, No. 966 Hengyu Road, Jinan, Fuzhou City, Fujian Province, China; ^2^Fujian Children's Hospital, No. 966 Hengyu Road, Jinan, Fuzhou City, Fujian Province, China; ^3^Fujian Maternity and Child Health Hospital, Affiliated Hospital of Fujian Medical University, No. 18 Daoshan Road, Gulou District, Fuzhou City, Fujian Province, China

## Abstract

Exploring the role of neuropeptides in the communication between monocyte subtypes facilitates an investigation of the pathogenesis of Kawasaki disease (KD). We investigated the patterns of interaction between neuropeptide-associated ligands and receptors in monocyte subpopulations in KD patients. Single-cell analysis was employed for the identification of cell subpopulations in KD patients, and monocytes were classified into 3 subpopulations: classical monocytes (CMs), intermediate monocytes (IMs), and nonclassical monocytes (NCMs). Cell-cell communication and differential analyses were used to identify ligand-receptor interactions in monocytes. Five neuropeptide-related genes (*SORL1*, *TNF*, *SORT1*, *FPR2*, and *ANXA1*) were involved in cell-cell interactions, wherein FPR2, a neuropeptide receptor, was significantly highly expressed in KD. Weighted gene coexpression network analysis revealed a significant correlation between the yellow module and FPR2 (*p* < 0.001, CC = 0.43). Using the genes in the yellow module, we constructed a PPI network to assess the possible functions of the FPR2-associated gene network. Gene set enrichment analysis showed that increased FPR2 levels may be involved in immune system regulation. FPR2 in CMs mediates the control of inflammation in KD. The findings of this study may provide a novel target for the clinical treatment of KD.

## 1. Introduction

Kawasaki disease (KD) is an acute, self-limiting disease that is characterized by systemic vasculitis and is the leading cause of acquired heart disease in children in developed countries [1, 2]. KD occurs mainly in children who are younger than 5 years and is less common in older children [3]. The prevalence of KD varies among different countries; for example, in Japan, the prevalence of KD among 5-year-old children is approximately 1% [4]. Currently, the exact classification and etiology of KD remain highly debated and controversial [5], although the etiopathogenesis of KD is attributed to a combination of genetic factors and dysfunctional immune responses to multiple antigens. Research into the mechanism of action of the immune response thus constitutes an important breakthrough in the diagnosis and treatment of KD [6].

Human monocyte subpopulations possess a wide range of complex functions. Different monocyte subpopulations perform diverse functions and are associated with multiple inflammatory conditions and diseases, including obesity, atherosclerosis, chronic obstructive pulmonary disease, lung cancer, and Alzheimer's disease [7, 8]. As evinced by the monocyte count, intermediate monocytes (IMs) potentially play a significant role in intravenous immunoglobulin (IVIG) resistance to the treatment of KD in children [9]. However, the functions of activated monocyte subpopulations are usually difficult to define, likely due to the heterogeneity of monocytes and the significant functional overlap between subpopulations [10]. Therefore, exploring the interaction of monocyte subtypes in KD could provide important insights to facilitate targeted therapy of monocytes to treat this bottleneck in KD.

Neuropeptides play an important role in the regulation of neuroendocrine processes and modulate many pathophysiological processes [11, 12], including in cell-cell interactions [13, 14]. The levels of various neuropeptides change during the course of KD [15, 16]. Therefore, we speculated that neuropeptides may mediate monocyte interactions in KD. However, the potential role of neuropeptides in KD, particularly in monocyte interactions, has not been investigated.

The development of single-cell RNA-seq (scRNA-seq) has facilitated precise investigations into the varying functions of different monocyte subpopulations. With the advent of bioinformatic techniques, deeper mechanisms and potential biomarkers have been identified from the analysis of scRNA-seq data [17, 18]. Weighted gene coexpression network analysis (WGCNA) can help identify coexpressed gene modules from gene expression data [19, 20]. Furthermore, correlation analysis can identify modules and genes that are significantly associated with clinical features. WGCNA has been used to identify key transcription factors that predict response to initial immunoglobulin therapy in acute KD [21]. Therefore, we believe that in combination with scRNA-seq analysis and bioinformatic techniques, WGCNA can facilitate investigations into the role of neuropeptide-related genes in the monocyte-mediated pathogenesis of KD. This study is aimed at improving the current understanding of KD monocyte subpopulation interactions, which can provide novel biomarkers for the diagnosis and treatment of KD.

## 2. Materials and Methods

### 2.1. Acquisition of Single-Cell Data and Quality Control

We used “Kawasaki Disease” as the keyword to retrieve KD-related single-cell datasets in the GEO database that were screened based on the following inclusion criteria to include datasets that (1) comprised single-cell transcriptomic data, (2) contained data from at least two KD patients, and (3) included pretreatment KD samples. The GSE152450 dataset, which met the abovementioned inclusion criteria, was included in our study [2, 22]. The GSE152450 dataset contains transcriptomic data of 8,880 mononuclear cells obtained from the peripheral blood samples of two KD pediatric patients and two healthy controls. After removing the data of “Multiplet” and “Undetermined” cells, the remaining data from 8,085 cells were further analyzed to determine data quality based on the inclusion of cells with at least 200 genes and of genes that were expressed in at least 3 cells. Finally, data from 7,271 cells were included in the subsequent single-cell analyses and for the evaluation of cell-cell communication. All included cells were treated for debatching effects.

### 2.2. Analysis of Single-Cell Data and Cell Annotation

The raw gene expression matrix of single cells was transformed into Seurat objects using Seurat (version 4.0.4). The gene expression matrix was normalized after quality control. Genes with high variability were identified and filtered using the FindVariableFeatures function and applying default parameters. Principal component analysis (PCA) of these highly variable genes was used to reduce the dimensionality to extract the expression features of each cell. Based on the inflection points on the elbow plot, we selected the principal components (PCs) that contained most of the original information and included these PCs in a clustering analysis (*p* < 0.05). The uniform manifold approximation and projection (UMAP) algorithm was used for dimensionality reduction to visualize cell clustering based on the selected PCs [23]. We identified the marker genes for each cell cluster using the FindAllMarkers function and demonstrated the top 10 marker genes in each cell cluster by using a heat map. The selection of marker genes for cell cluster annotation was performed according to the previously described protocols and instructions on the CellMarker website (http://biocc.hrbmu.edu.cn/CellMarker/index.jsp) [24–26].

### 2.3. Identification of Neuropeptide-Related Marker Genes

We included 87 neuropeptide-related molecules that were obtained through searches of literature reviews in PubMed and in the genecard database. Neuropeptide-associated genes encode neuropeptides and receptors. After cross-tabulation analysis to identify neuropeptide-related ligand and receptor molecules in cell clusters with specific marker genes, we could identify genes that are functionally related to neuropeptides and possibly play a role in cell-cell communication between these cell clusters.

### 2.4. Analysis of Ligand-Receptor Interactions in Cell-Cell Communication

CellPhoneDB was used to analyze cell-cell communication. Single-cell gene expression matrices were calculated as input files to predict ligand-receptor interaction pairs among cells [27, 28]. We further screened neuropeptide-associated cluster marker genes from these predicted ligand-receptor pairs, which were then included in the subsequent analysis.

### 2.5. Weighted Gene Coexpression Network Analysis in Monocytes

To further explore the regulatory role of neuropeptides in monocytes, monocyte expression matrices that were extracted from single-cell expression matrices were evaluated through WGCNA [19, 29] using the WGCNA package in R (version 1.70.3), and an approximate scale-free topology network was constructed for the analysis of module functionality [19, 29]. First, we used the pickSoftThreshold function to identify the soft-thresholding powers that satisfy the criterion of approximate scale-free topology. Unsigned network and topological overlap measure (TOM) were chosen to calculate the intergene correlation between genes. The genes in the matrix were further hierarchically clustered to construct clustering trees based on the TOM values. The clustering tree was segregated into several clusters using a predefined fixed height. PCA was used to identify the Epigen gene to represent the overall level of gene expression within each module. Epigen gene significance and Pearson correlation analysis of the desired factors were used to screen for modules associated with the interesting factors. We selected modules based on Pearson correlation coefficients > 0.35 for further analysis.

### 2.6. Protein-Protein Interaction Network Analysis

To explore the function of these modules, we constructed a protein-protein interaction (PPI) network for the genes included within these modules that were then entered into the STRING database (https://www.string-db.org/) for protein matching and network construction [29, 30]. Depending on the number of molecules in the network and the size of the network, we set the appropriate minimum required interaction score and removed isolated nodes that were not connected to other nodes. Finally, we described and visualized the protein functions within the constructed PPI networks.

### 2.7. Gene Set Enrichment Analysis

Gene set enrichment analysis (GSEA) was used to analyze the function and potential enrichment pathways of neuropeptide-related molecules in cells and was set up as follows: number of random sample interchanges, *n* = 1000, with at least 10 genes and up to 500 genes in each gene set. The “c2.cp.v7.2.symbols.gmt [Curated]” gene set from MSigDB collections (https://www.gsea-msigdb.org/gsea/msigdb/) was used as a reference gene set for GSEA [31]. The R package http://org.hs.eg/.db was used for the conversion of gene ID, and clusterProfiler was used for calculating the significance of the enriched pathway gene sets [32]. The conditions for significant enrichment were a false discovery rate (FDR) < 0.25 and adjusted *p* < 0.05.

### 2.8. Analysis of Differential Gene Expression between HI and KD

R (version 4.0.2) was used for the majority of statistical analysis and to construct the graphical plots. We used DESeq2 (version 1.20) to analyze differential gene expressions between the healthy individual (HI) and KD groups [33]. To explore the role of a monocyte (CM) subpopulation in KD pathology, we separately analyzed the differential gene expressions in the CM subpopulations. Cellular gene expression matrices in the same sample were summed according to gene to generate a bulky RNA-seq-like dataset. Thus, we could evaluate and compare the ploidy changes in genes between the HI and KD groups and determine the *p* values of differentially expressed genes (DEGs), which were used to further screen for neuropeptide-related ligands and/or receptors. *p* < 0.05 was considered statistically significant unless stated otherwise. All data were saved to enable replication of the analysis.

## 3. Results

### 3.1. Cellular Heterogeneity and Clustering Results

**Figure 1:**
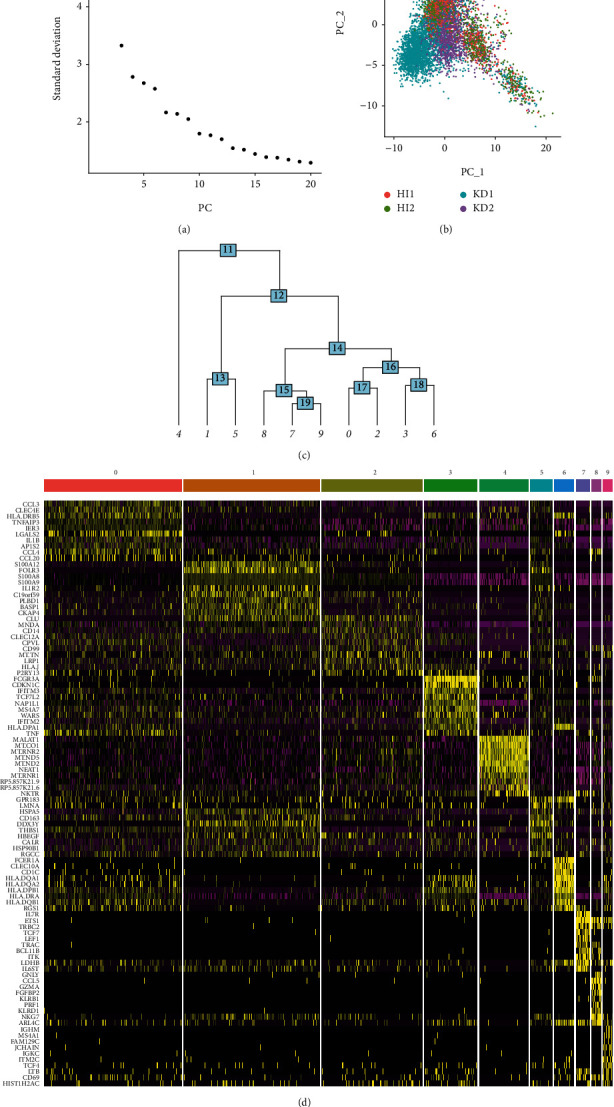
Downscaling and clustering processes for the data of single-cell transcripts from the GSE152450 dataset. (a) The elbow plot shows the inflection point between PCs 10 and 13, indicating that selecting these PC numbers in the subsequent downscaling analysis would help retain most of the original data information. (b) PC1 and PC2 obtained during PCA and the dimensionality reduction results of the single-cell data for the 4 samples are presented here. Different samples are indicated by different colors. (c) Herein, 13 PCs were selected to construct the clustering tree, and the results show 10 cell clusters. (d) In each of these 10 clusters, the top 10 cluster marker genes, along with their expression levels, are represented in the heat map.

After rigorous quality screening of cells, we selected 2,968 and 4,303 cells from the HI and KD groups, respectively, for further analyses. The results of quality control performed using the data from single-cell sequencing are shown in Supplementary Figures 1A–1C. ANOVA revealed the top 10 highly variable genes in single cells (Supplementary Figure 1D). Elbow plots showed that the forward 13 PCs contained most of the gene information that was expressed by the cells (Figure 1(a)). The PCA plot using principal component 1 (PC1) and principal component 2 (PC2) showed that the single-cell data of HI1 and HI2 overlapped after downscaling, whereas those of KD1 and KD2 only overlapped partially after downscaling (Figure 1(b)). This suggests that there may be differences in cell composition between KDs and HIs. The abovedescribed 13 PCs were used to construct a clustering tree wherein 10 clusters were identified (Figure 1(c)) along with their marker genes, and the expression of the top 10 marker genes in each cluster is presented as a heat map (Figure 1(d)).

### 3.2. Cell Annotation and Identification of Three Monocyte Subpopulations

**Figure 2:**
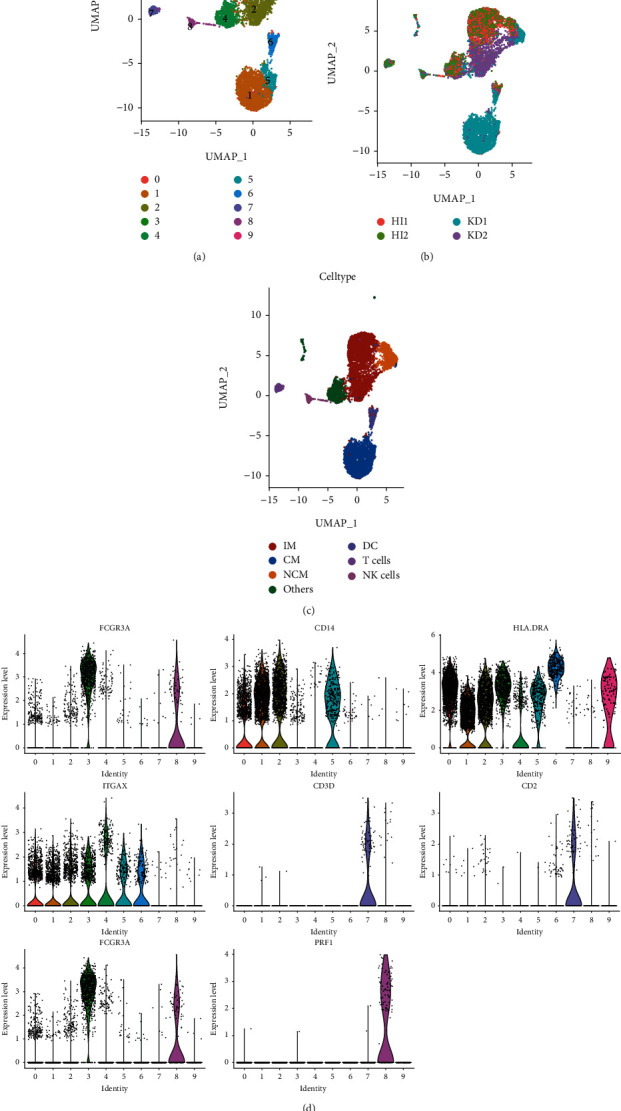
Cellular annotation of molecules and clusters. (a) UMAP represents the 10 cell clusters, and different colors indicate different clusters. (b) The sources of cells corresponding to the 10 clusters are labeled in the UMAP. (c) Cell clusters were accurately annotated into 6 cell types. Among them, NCM, IM, and CM are subtypes of monocytes. (d) The 8 molecules used for cellular annotation and their expression in each of the 10 clusters are shown.

First, we performed cell annotation of the 10 cell clusters and visualized the single-cell distribution of these 10 clusters and the 4 samples by using the UMAP of the 13 selected PCs (Figures 2(a) and 2(b)). Next, we annotated these 10 cell clusters with 8 marker genes, namely, *FCGR3A*, *CD14*, *HLA-DRA*, *ITGAX*, *CD3D*, *CD2*, *FCGR3A*, and *PRF1*, and annotated a total of 6 cell types. Human monocytes were annotated into 3 categories based on the expression of CD16 (FCGR3A) and CD14. CD14+CD16- monocytes were annotated as monocytes (CMs), CD14+CD16+ monocytes were annotated as intermediate monocytes (IMs), and CD14-CD16+ monocytes were annotated as nonclassical monocytes (NCMs). The other 3 cell types identified were dendritic cells (DCs; HLA-DRA+, ITGAX+, CD14-, and FCGR3A-), NK cells (FCGR3A+ and PRF1+), and T cells (CD3D+ and CD2+). The UMAP to visualize these 6 cell types is presented in Figure 2(c).  Figure 2(d) shows the expression of the 8 marker genes in the 10 cell clusters. To assess the role of neuropeptide-related molecules in monocyte subpopulations, the expression matrices of monocytes were extracted for further analysis.

### 3.3. The Interactions between Ligand-Receptor Pairs during Cell-Cell Communication

**Figure 3:**
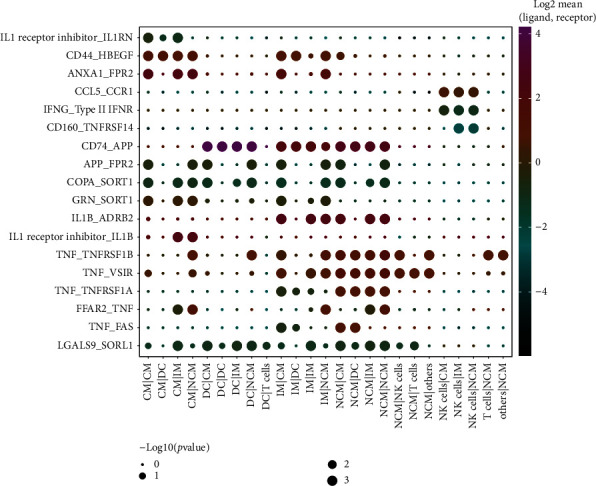
Predicted results of neuropeptide-related ligand-receptor interactions between the 6 identified cell types. The vertical axis shows the interacting ligand-receptor molecule pairs, and the horizontal axis shows the two pairs of interactions between the 6 cell types. The dots in the graph represent the results of the correlation analysis between the cell-cell interaction pairs and the corresponding ligand-receptor interaction pairs. The size and color of the dots denote the significance and strength, respectively, of the ligand-receptor interaction.

We analyzed cell-cell communication among the 6 annotated cell types. And 144 ligand-receptor interaction pairs among these 7 cell populations were found. Of these ligand-receptor pairs, 5 neuropeptide-related mRNAs were identified: *SORL1*, *TNF*, *SORT1*, *FPR2*, and *ANXA1*. The correlation heat map presented in Figure 3 shows the relationship between the ligand-receptor pairs containing the neuropeptide-related genes (Figure 3). *FPR2* and *ANXA1* have a significantly positive activating effect in all 3 monocyte subtypes. DCs can regulate the function of monocytes by releasing APP to bind the formyl-peptide receptor 2 (FPR2). In addition, *SORL1*, *TNF*, and *SORT1* played a role in monocyte regulation through a ligand-receptor mechanism of action.

### 3.4. WGCNA Reveals Gene Modules Related to Neuropeptides

**Figure 4:**
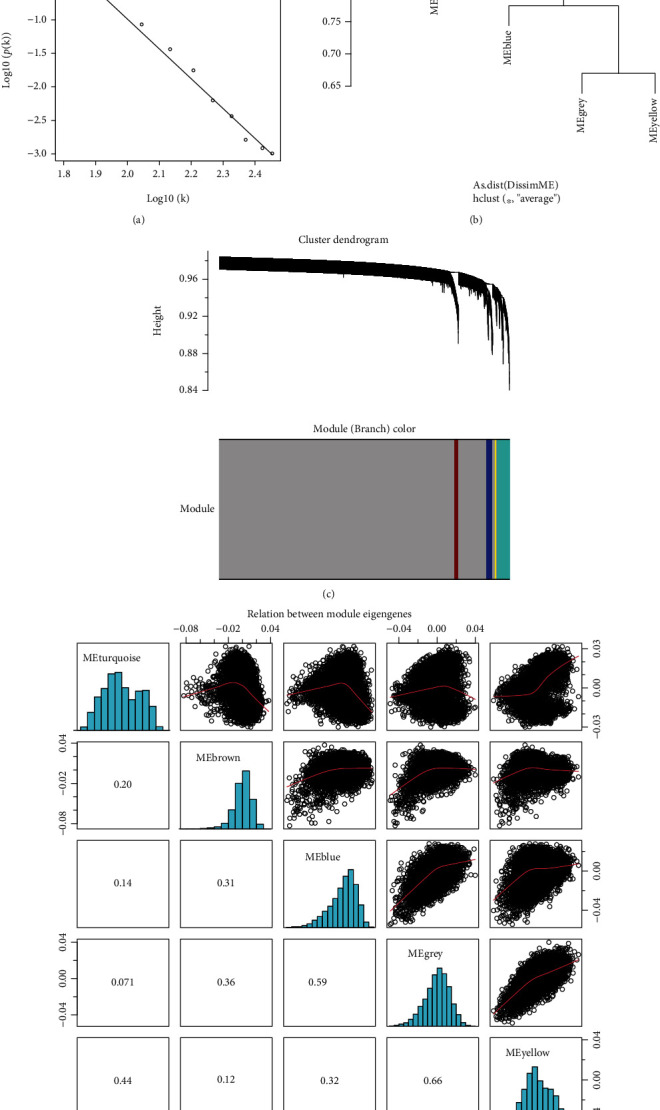
WGCNA of monocyte transcriptional data. (a) When the power is 1, *R*^2^ is 0.99, indicating that a scale-free network was successfully constructed using single-cell transcript data. (b, c) Based on the selected networks, clustering trees were constructed. Employing the static shear tree approach, 5 modules were acquired. (d) Correlation analysis demonstrating the relationship between Epigen genes in the 5 gene modules.

**Figure 5:**
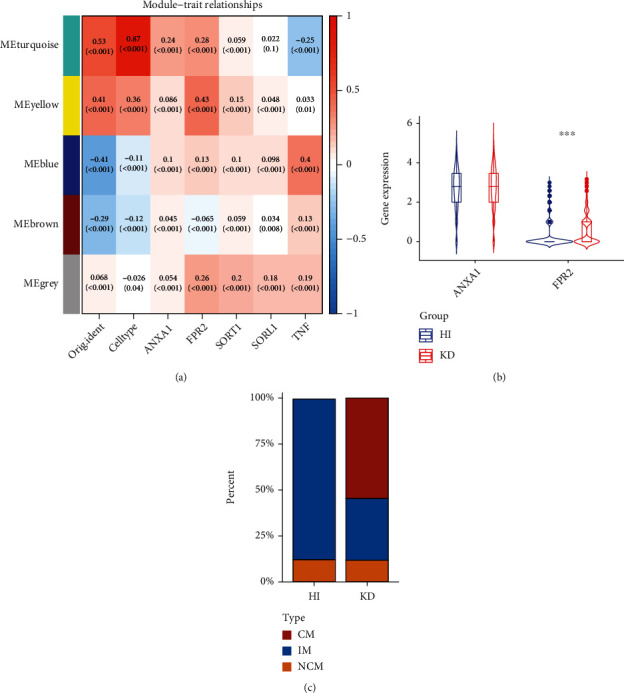
Relationship between the 5 gene modules and desired indicators determined using WGCNA. (a) Heat map representing the correlation between the 5 modules and 5 neuropeptide-related ligands/receptors obtained from the results of ligand-receptor interaction analysis. (b) FPR2 expression in monocytes differs between the HI and KD groups in these 5 neuropeptide-related ligands/receptors. (c) Comparison between the HI and KD groups for the proportion of the 3 monocyte subtypes. The proportion of CMs in the KD group was significantly higher than that in the HI group.

WGCNA was used to identify gene modules related to the 5 neuropeptide-related genes (*SORL1*, *TNF*, *SORT1*, *FPR2*, and *ANXA1*) that were identified in this study. First, the scale-free network was successfully constructed when the soft threshold power was 1 (*R*^2^ = 0.99, Figure 4(a)). Then, a clustering tree was constructed for the network, and the static shear tree method was used to obtain 5 modules (Figures 4(b) and 4(c)).  Figure 4(d) shows the relationship between module eigengenes. The correlation heat map in Figure 5(a) shows a significant correlation between *FPR2* and the yellow module (*p* < 0.001, CC = 0.43). Analysis of gene differences in CMs showed the differences in the expression levels of *ANXA1* and *FPR2* between the HI and KD groups (Figure 5(b)). Furthermore, the expression of FPR2 in monocytes significantly differed between the HI and KD groups.  Figure 5(c) shows that the proportion of CMs in the KD group was significantly higher than that in the HI group. Moreover, Figure 5(a) shows that TNF levels were significantly correlated with the blue modules (*p* < 0.001, CC = 0.4). The yellow and turquoise modules were significantly correlated with both the sample sources and cell types. The results of correlation analysis between genes in the yellow module and the 5 neuropeptide-related molecules are presented in Supplementary Figure 2.

### 3.5. PPI Network Analysis

**Figure 6:**
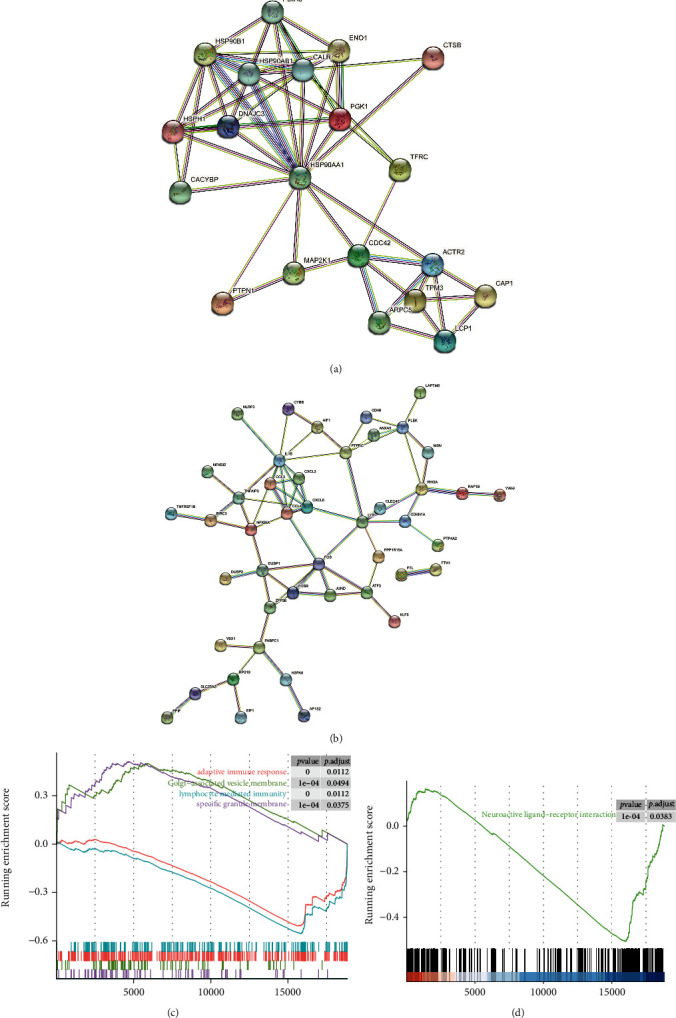
Protein-protein interaction networks in the yellow and blue modules and GSEA of FPR2. (a) A PPI network based on the genes in the yellow module was constructed to observe the intramodule gene interactions. (b) PPI network based on the genes in the blue module. (c) GSEA demonstrating the up- and downregulated GO pathways in cells with increased FPR2 expression. (d) GSEA demonstrating the downregulated KEGG pathway in cells with increased FPR2 expression.

To explore the possible roles of the modules that were being investigated, we constructed a PPI network for genes in the yellow, blue, and turquoise modules. Figures 6(a) and 6(b) present PPI networks constructed from the genes in the yellow and blue modules, respectively. In these networks, some pivotal genes, such as HSP90AA1 in the yellow module and CCL4 in the blue module, can be seen. In addition, the PPI network constructed from the genes in the turquoise module is presented in Supplementary Figure 3 and revealed the interactions between proteins in different modules.

### 3.6. Functional Analysis of FPR2 Using GSEA

To explore the function of FPR2 in cells, we used GSEA to analyze FPR2-enriched GO and KEGG pathways. Upregulated FPR2 was found to be enriched in the following GO pathways: Golgi-associated vesicle membranes and specific granule membranes. In contrast, FPR2 was downregulated in lymphocyte-mediated immunity, adaptive immune response, and neuroactive ligand-receptor interaction pathways, which suggests that increased FPR2 levels may participate in immune system regulation.

## 4. Discussion

Monocytes play a key role in inflammatory responses and are important immune cells in KD. However, due to heterogeneity and functional overlap among the monocyte subpopulations, monocyte subsets prove challenging to clearly define and delineate. Moreover, the role of neuropeptides as critical mediators in neuroendocrine regulation remains unclear in KD pathology. This study investigated the patterns of interaction between neuropeptide-associated ligands and receptors in monocyte subpopulations in KD patients. FPR2 in CMs is involved in the control of inflammation in KD.

Based on the screening criteria, the GSE152450 dataset containing transcriptomic data from healthy controls and children with acute KD was used in this study. First, cells were downscaled, clustered, and annotated according to gene expression. Then, according to the previous classification of human monocytes, we classified monocytes into 3 subgroups (namely, CMs, IMs, and NCMs) based on CD14 and CD16 expression. We analyzed monocyte subpopulation-specific interactions between neuropeptide-related ligand-receptor molecules. Using differential analysis, we identified *FPR2* as a gene that encodes a CM-specific neuropeptide receptor and further used WGCNA to discern that the yellow module in the coexpression network correlated with *FPR2* expression. The genes in the yellow module were used to construct a PPI network to assess the possible functions of the *FPR2*-associated gene network. Finally, GSEA was used to identify the GO and KEGG pathways that were altered when FPR2 expression levels increased.

Monocytes are important inflammatory regulators that play diverse roles in vasculitis as well as in immune responses [34, 35]. Based on the CD14 and CD16 expression patterns, human monocytes can be classified into 3 major subpopulations—CMs, IMs, and NCMs. CMs are usually phagocytes without inflammatory activity; NCMs can display inflammatory features; and IMs may be transitional cells with both phagocytic and inflammatory functions [36]. A study noted that CMs exhibit a more proinflammatory phenotype during antimicrobial response, whereas another study reported that CMs are usually phagocytes without inflammatory properties [36, 37]. In the present study, the proportion of CMs was significantly higher in the KD group than in healthy controls, suggesting that CM-mediated proinflammatory responses or phagocytosis may be significantly increased in KD.

Formyl-peptide receptors (FPRs) play crucial roles in various pathophysiological conditions, including inflammation control, tissue repair, and angiogenesis [38, 39]. Among these, *FPR2* is the most promising member, as it can recognize various lipids, proteins, and neuropeptides and act as a “double-sided” molecule in both pro- and anti-inflammatory responses, depending on the binding of different agonists [40–42]. Involvement in many normal physiological responses and in the etiopathogenesis of diseases, such as RA, makes FPR2 a very attractive therapeutic target [43]. Vasoactive intestinal peptide (VIP) can effectively improve corneal inflammation by influencing FPR2 pathway activation [44]. FPR2 agonists can help improve the healing process after myocardial infarction, thus providing an innovative option for therapy [45]. Annexin A1 (ANXA1) is an important mediator that regulates glucocorticoid expression, and FPR2 mediates inflammatory response under *ANXA1* regulation [46–48]. In this study, FPR2 expression and CM counts were significantly higher in the KD group than in healthy controls, suggesting that CMs may play a crucial role in the development of FPR2-mediated inflammation. Furthermore, cell-cell communication analysis demonstrated that ANXA1-FPR2 interactions occur during cellular interactions between multiple cell types and CMs. The main features of acute KD include signaling pathways of the innate immune system [49]. GSEA suggests that elevated levels of FPR2 may be involved in the regulation of homeostasis within the immune system.

However, the present study has certain limitations. First, KD staging was not specifically studied in this research. Fortunately, the GSE152450 collection included transcriptome data from 8880 mononuclear cells collected from peripheral blood samples. Because all of the cells in this study were debatched, the differences between the HI and KD groups could be compared more accurately. Second, we identified some interacting neuropeptide-related molecules between monocytes mainly by using public datasets and bioinformatic techniques; however, these data were acquired from a dataset with sample-size constraints. Therefore, validation of the clinical samples and experiments is needed. In addition, we only analyzed monocytes, which somewhat limits the assessment of the entire immune system in patients with KD that should be examined in further studies to identify the key genes in the various immune cells causing KD.

## 5. Conclusion

The neuropeptide receptor FPR2 in CMs plays a role in the regulation of inflammation in KD. The findings of this study will help us elucidate the mechanisms of action of monocyte subpopulations in KD and pave the path for a new direction in the clinical treatment of KD.

## Data Availability

The datasets are available from the GSE152450 dataset.

## Abbreviations

ANXA1: Annexin A1
CM: Classical monocytes
FPRs: Formyl-peptide receptors
GC: Glucocorticoids
IM: Intermediate monocytes
IVIG: Intravenous immunoglobulin
KD: Kawasaki disease
NCM: Nonclassical monocytes
PCs: Principal components
scRNA-seq: Single-cell RNA-seq
TOM: Topological overlap measure
UMAP: Uniform manifold approximation and projection
VIP: Vasoactive intestinal peptide
WGCNA: Weighted gene coexpression network analysis.
