# Supplementary material for: Single-Cell Analysis Reveals the Role of the Neuropeptide Receptor FPR2 in Monocytes in Kawasaki Disease: A Bioinformatic Study
Source: Dis Markers. 2022 Jun 1;2022:1666240. doi: 10.1155/2022/1666240 (PMC9177323; doi:10.1155/2022/1666240)
Supplement: Supplementary Materials — All supplementary figures and descriptions are available in the “Supplementary figures” file. [file 1666240.f1.docx]

**Supplementary Figure Legends**


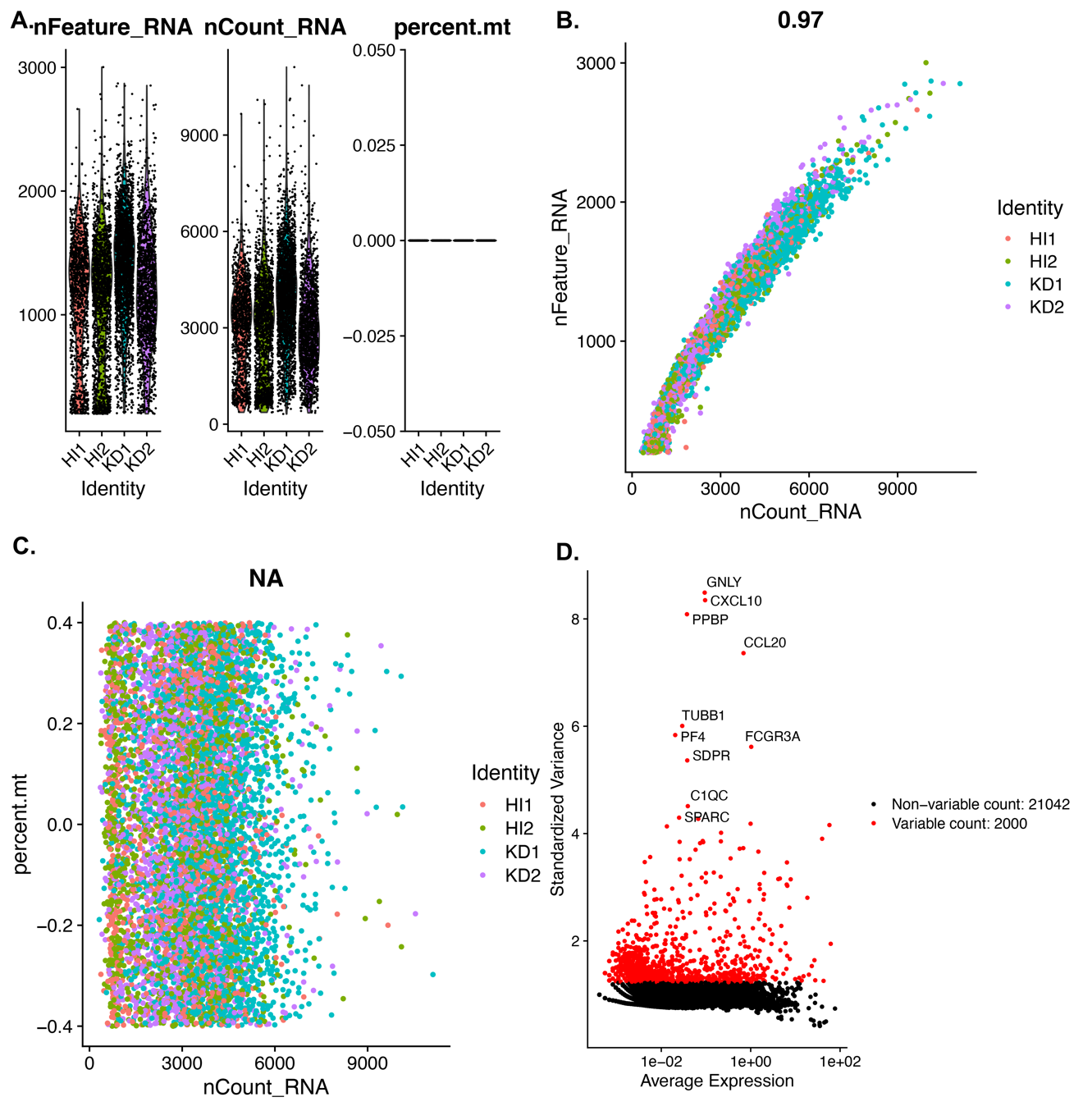


**Supplementary Figure 1. Quality control of single-cell transcript data from the GSE152450 dataset.**

A. The results of quality control of single-cell transcript data in the four samples after data filtering. Violin plots show the distribution of unique feature counts, total RNA counts, and the percentage of mitochondrial genes in the cells.

B. Scatter plot showing the relationship between unique feature counts and total RNA counts. The results demonstrate a linear correlation, indicating a strong relationship between them.

C. The scatter plot of the percentage of total RNA counts and mitochondrial genes shows that the percentage of mitochondrial genes in each cell is less than 5% and shows a random distribution that is independent of total RNA counts.

D. ANOVA revealed a highly variable characterization of the top 10 genes in single cells.


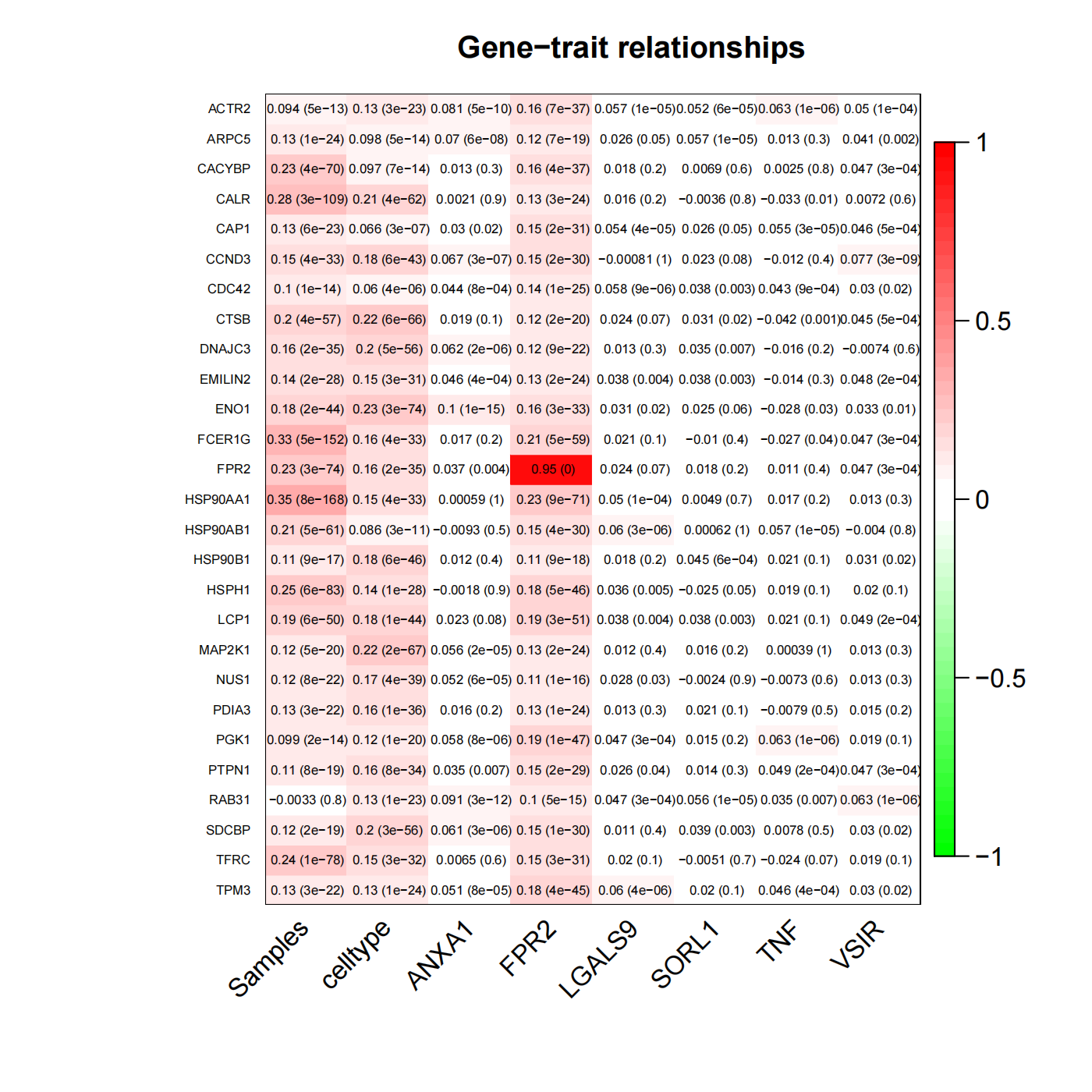


**Supplementary Figure 2. Correlation analysis between the five neuropeptide-related ligands/receptors and the genes in the yellow module.** Red color indicates positive correlation and green color indicates negative correlation.


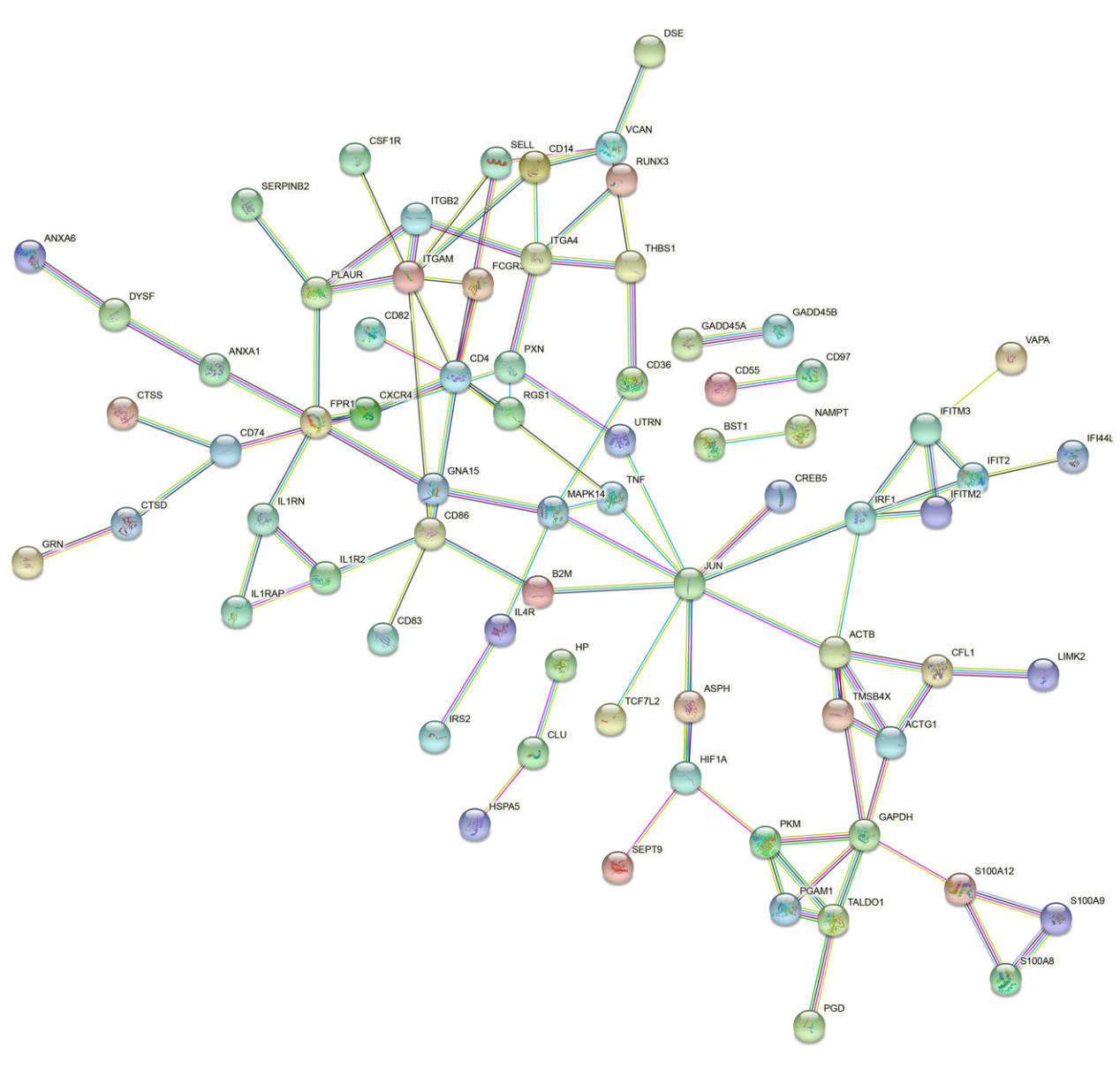


**Supplementary Figure 3. PPI network constructed using the genes in the turquoise module.**
